# The genome sequence of *Gymnosoma rotundatum* (Linnaeus, 1758), a parasitoid ladybird fly

**DOI:** 10.12688/wellcomeopenres.17782.1

**Published:** 2022-03-21

**Authors:** Matthew Smith, Natural History Museum Genome Acquisition Lab, Darwin Tree of Life Barcoding collective, Wellcome Sanger Institute Tree of Life programme, Wellcome Sanger Institute Scientific Operations: DNA Pipelines collective, Tree of Life Core Informatics collective, Darwin Tree of Life Consortium

**Affiliations:** 1 Independent Researcher, Reading, UK

**Keywords:** Gymnosoma rotundatum, genome sequence, chromosomal, Diptera

## Abstract

We present a genome assembly from an individual male *Gymnosoma rotundatum *(Arthropoda; Insecta; Diptera; Tachinidae). The genome sequence is 779 megabases in span. The majority of the assembly (97.07%) is scaffolded into six chromosomal pseudomolecules, with the X sex chromosome assembled.

## Species taxonomy

Eukaryota; Metazoa; Ecdysozoa; Arthropoda; Hexapoda; Insecta; Pterygota; Neoptera; Endopterygota; Diptera; Brachycera; Muscomorpha; Oestroidea; Tachinidae; Phasiinae; Gymnosomatini; Gymnosoma; *Gymnosoma rotundatum* (Linnaeus, 1758) (NCBI:txid569046).

## Background

The Tachinid flies (Diptera: Tachinidae) are one of the largest families of flies. The entire family are parasitic, with the larvae developing as internal parasites in a range of hosts, mostly insects. *Gymnosoma rotundatum* (Diptera: Tachinidae) is a small, 5-6-mm-long fly with a dark thorax dusted with gold in males, and a globular red or orange abdomen decorated with dark markings along the midline. This shape and colouration has given rise to the use of the name "ladybird flies" as a common name for various *Gymnosoma* species.

*Gymnosoma rotundatum* is known from Britain and Ireland ( Belshaw, 1993). In Ireland, the species has only been recorded from a few localities in southern Ireland, with the most recent record from County Kerry in 2015. In Britain, the species has historically been regarded as rare, and was accorded Red Data Book 3 status by Falk (1991). Morris (1997) summarised the known British records and distribution of the species up to 1996, noting that *G. rotundatum* appeared to be "seemingly confined to a narrow corridor from the West Sussex coast through Surrey and parts of North Hampshire". *Gymnosoma rotundatum* appears to be one of the species benefiting from the warming climate in the UK, and since 1996 it has been increasingly recorded away from its restricted historical range. It is now known from a large number of sites in central southern and south-east England, with a few recent records from East Anglia. 

*Gymnosoma rotundatum* is a parasite of Shieldbugs (Hemiptera: Pentatomidae), though specific host details are limited. Tschorsnig & Herting (1994) only cite "Pentatomidae" and Belshaw (1993) lists *Palomena* spp. as a host, although there are no confirmed British rearing records. Adult flies are on the wing from late April until early October, with records peaking in August. The species is most often recorded from warm dry sites, where it visits a range of open shallow flowers such as Hogweed ( *Heracleum sphondylium*), Yarrow ( *Achillea millefolium*) and Mayweeds ( *Tripleurospermum* sp.).

## Genome sequence report

**Figure 1.**
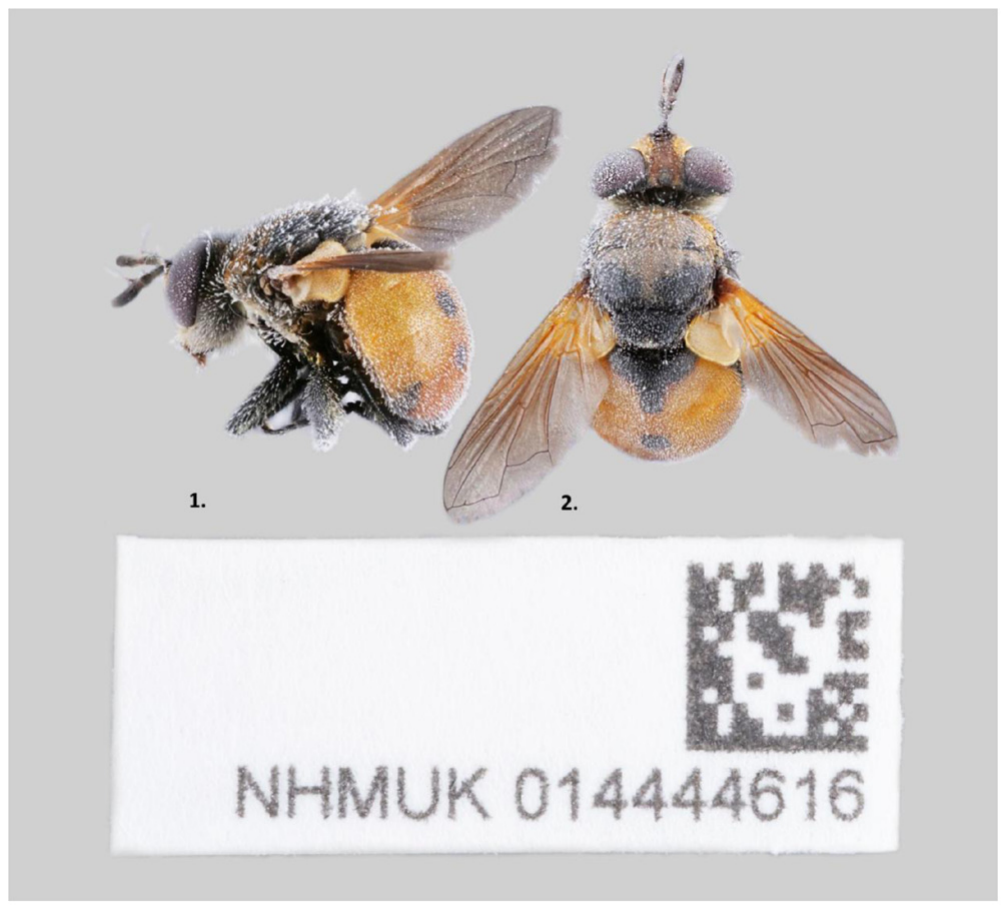
Images of the Gymnosoma rotundatum specimen, taken during preservation and processing. Left, lateral view; right, dorsal view.

The genome was sequenced from a single male *G. rotundatum* ( Figure 1) collected from Hartslock Reserve, Oxfordshire, UK (latitude 51.511263, longitude -1.112222). A total of 31-fold coverage in Pacific Biosciences single-molecule long reads and 32-fold coverage in 10X Genomics read clouds were generated. Primary assembly contigs were scaffolded with chromosome conformation Hi-C data. Manual assembly curation corrected 191 missing/misjoins and removed 3 haplotypic duplications, reducing the assembly size by 0.11% and the scaffold number by 23.88%, and increasing the scaffold N50 by 14.22%.

**Figure 2.**
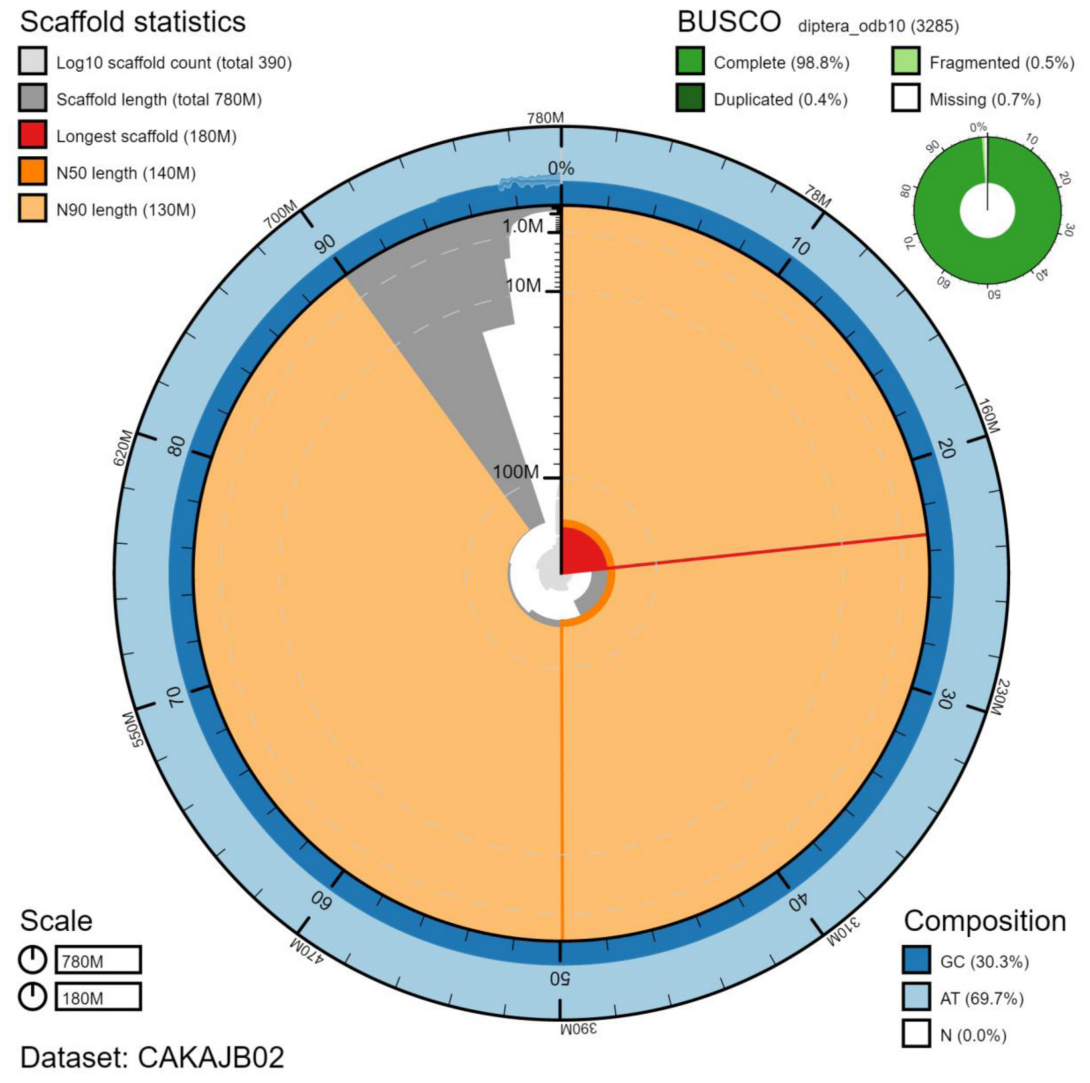
Genome assembly of *Gymnosoma rotundatum*, idGymRotn1.2: metrics. The BlobToolKit Snailplot shows N50 metrics and BUSCO gene completeness. The main plot is divided into 1,000 size-ordered bins around the circumference with each bin representing 0.1% of the 779,146,119 bp assembly. The distribution of scaffold lengths is shown in dark grey with the plot radius scaled to the longest scaffold present in the assembly (182,003,241 bp, shown in red). Orange and pale-orange arcs show the N50 and N90 scaffold lengths (137,798,182 and 132,556,942 bp), respectively. The pale grey spiral shows the cumulative scaffold count on a log scale with white scale lines showing successive orders of magnitude. The blue and pale-blue area around the outside of the plot shows the distribution of GC, AT and N percentages in the same bins as the inner plot. A summary of complete, fragmented, duplicated and missing BUSCO genes in the diptera_odb10 set is shown in the top right. An interactive version of this figure is available at https://blobtoolkit.genomehubs.org/view/idGymRotn1.2/dataset/CAKAJB02/snail.

The final assembly has a total length of 779 Mb in 392 sequence scaffolds with a scaffold N50 of 137.8 Mb ( Table 1). The majority, 97.07%, of the assembly sequence was assigned to 6 chromosomal-level scaffolds, representing 5 autosomes (numbered by sequence length), and the X sex chromosome ( Figure 2– Figure 5; Table 2). The X chromosome has been identified based on half diploid coverage. There are a large number of unassigned scaffolds that may belong to X or Y, as we are uncertain whether the karyotype is X0 or XY. The assembly has a BUSCO v5.2.2 ( Manni *et al.*, 2021) completeness of 98.8% (single 98.3%, duplicated 0.4%) using the diptera_odb10 reference set. While not fully phased, the assembly deposited is of one haplotype. Contigs corresponding to the second haplotype have also been deposited.

**Table 1.** Genome data for *Gymnosoma rotundatum*, idGymRotn1.2.

*Project accession data*	
Assembly identifier	idGymRotn1.2
Species	*Gymnosoma rotundatum*
Specimen	idGymRotn1
NCBI taxonomy ID	569046
BioProject	PRJEB46301
BioSample ID	SAMEA7849381
Isolate information	Male, thorax (genome assembly), head (Hi-C)
*Raw data accessions*	
PacificBiosciences SEQUEL II	ERR6939227
10X Genomics Illumina	ERR6688431-ERR6688434
Hi-C Illumina	ERR6688430
*Genome assembly*	
Assembly accession	GCA_916610165.2
*Accession of alternate haplotype*	GCA_916610175.2
Span (Mb)	779
Number of contigs	623
Contig N50 length (Mb)	9.4
Number of scaffolds	392
Scaffold N50 length (Mb)	137.8
Longest scaffold (Mb)	182.0
BUSCO * genome score	C:98.8%[S:98.3%,D:0.4%],F:0.5%,M:0.7%,n:3285

*BUSCO scores based on the diptera_odb10 BUSCO set using v5.2.2. C= complete [S= single copy, D=duplicated], F=fragmented, M=missing, n=number of orthologues in comparison. A full set of BUSCO scores is available at https://blobtoolkit.genomehubs.org/view/idGymRotn1.2/dataset/CAKAJB02/busco.

**Figure 3.**
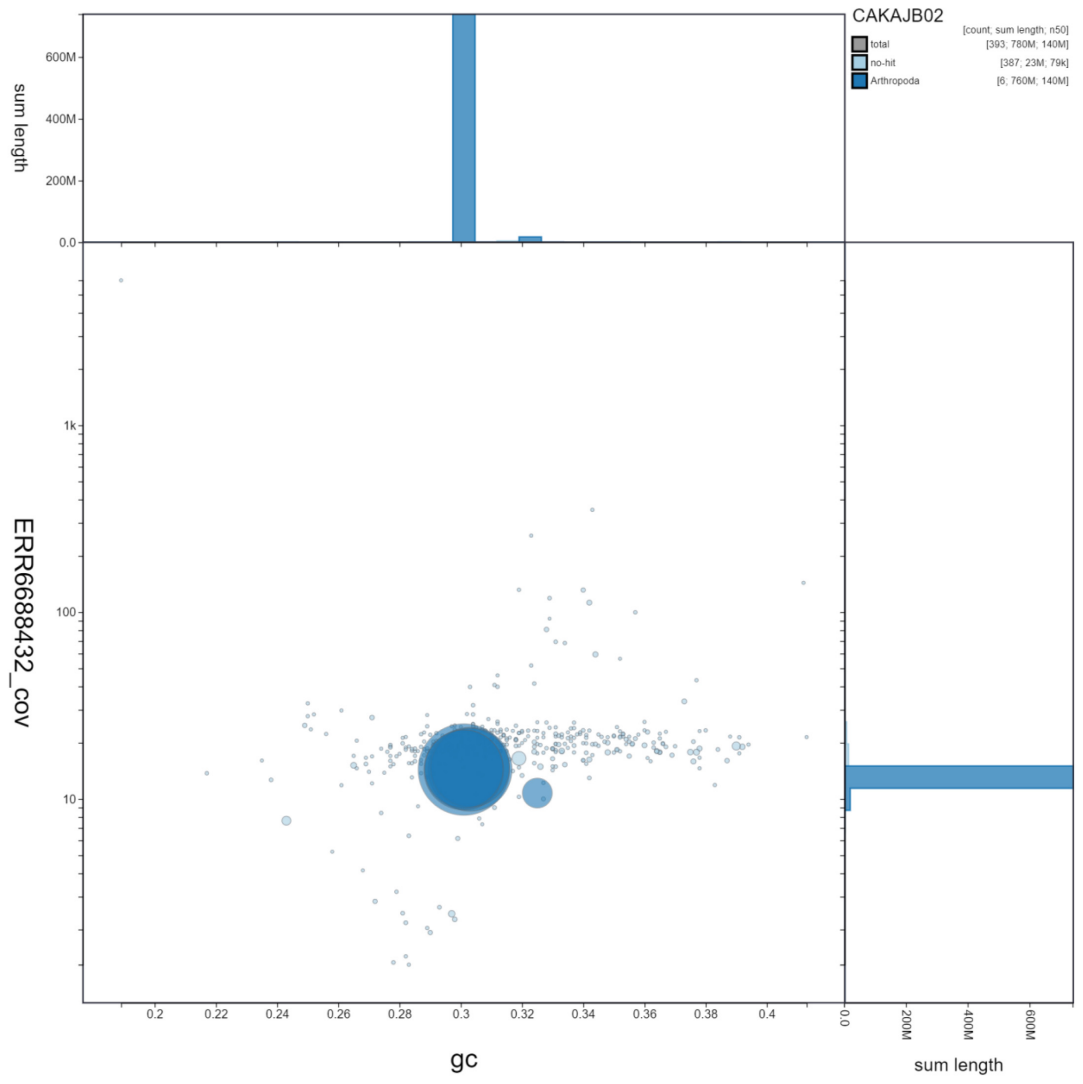
Genome assembly of *Gymnosoma rotundatum*, idGymRotn1.2: GC coverage. BlobToolKit GC-coverage plot. Scaffolds are coloured by phylum. Circles are sized in proportion to scaffold length. Histograms show the distribution of scaffold length sum along each axis. An interactive version of this figure is available at https://blobtoolkit.genomehubs.org/view/idGymRotn1.2/dataset/CAKAJB02/blob.

**Figure 4.**
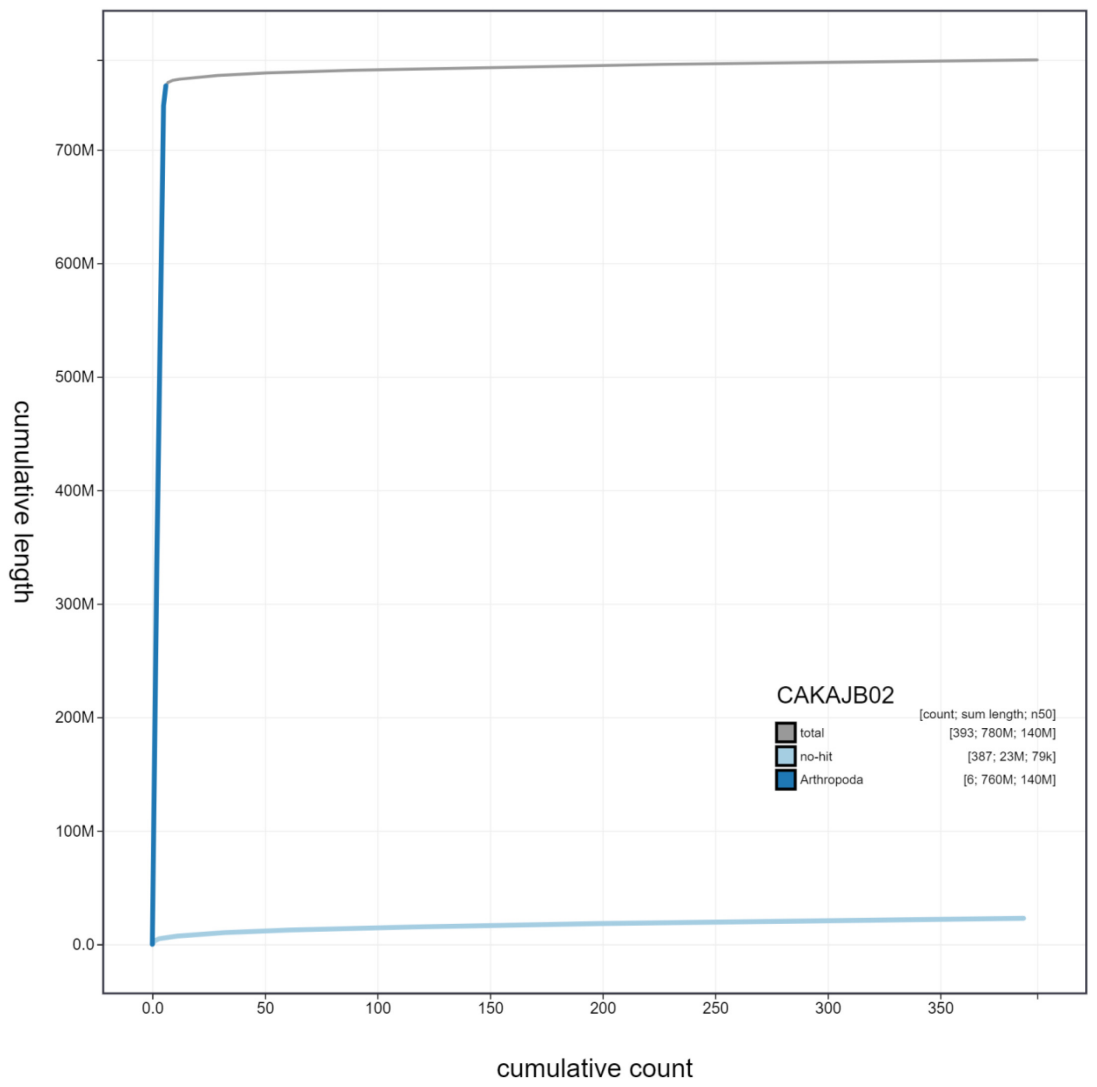
Genome assembly of *Gymnosoma rotundatum*, idGymRotn1.2: cumulative sequence. BlobToolKit cumulative sequence plot. The grey line shows cumulative length for all scaffolds. Coloured lines show cumulative lengths of scaffolds assigned to each phylum using the buscogenes taxrule. An interactive version of this figure is available at https://blobtoolkit.genomehubs.org/view/idGymRotn1.2/dataset/CAKAJB02/cumulative.

**Figure 5.**
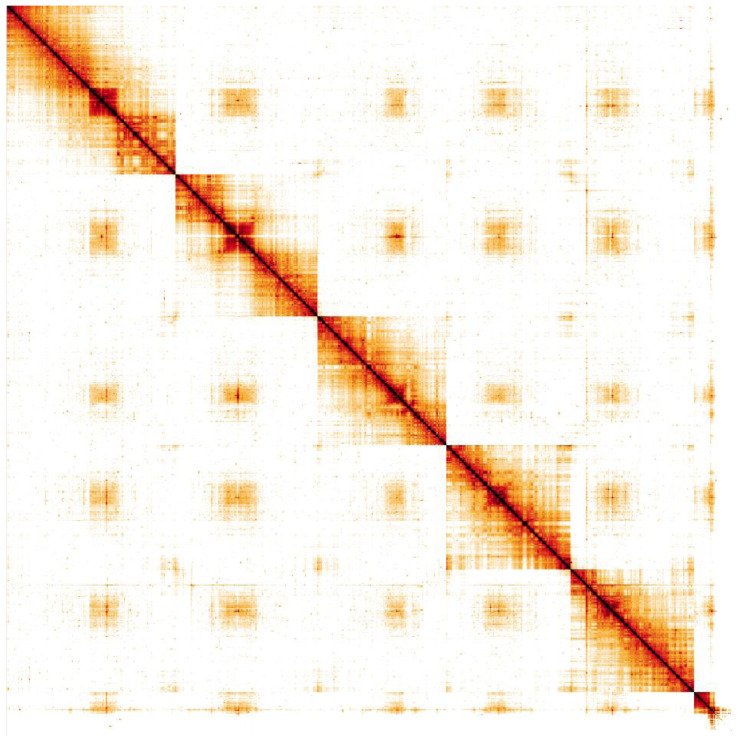
Genome assembly of *Gymnosoma rotundatum*, idGymRotn1.2: Hi-C contact map. Hi-C contact map of the idGymRotn1.2 assembly, visualised in HiGlass. Chromosomes are presented in order of size from left to right and top to bottom. An interactive version of this figure is available here.

**Table 2.** Chromosomal pseudomolecules in the genome assembly of *Gymnosoma rotundatum*, idGymRotn1.2.

INSDC accession	Chromosome	Size (Mb)	GC%
OU744336.1	1	182.00	30.1
OU744337.1	2	152.51	30.3
OU744338.1	3	137.80	30.1
OU744339.1	4	134.05	30.3
OU744340.1	5	132.56	30.1
OU744341.1	X	17.34	32.5
OU744342.1	MT	0.02	18.9
-	Unplaced	22.87	32.2

## Methods

### Sample acquisition and nucleic acid extraction

A male *G. rotundatum* (idGymRotn1) was collected from Hartslock Reserve, Oxfordshire, UK (latitude 51.511263, longitude -1.112222) by Matt Smith, independent researcher, who also identified the specimens. The specimens were collected from grassland using a net and snap-frozen in liquid nitrogen.

DNA was extracted at the Tree of Life laboratory, Wellcome Sanger Institute. The idGymRotn1 sample was weighed and dissected on dry ice with tissue set aside for Hi-C sequencing. Thorax tissue was disrupted using a Nippi Powermasher fitted with a BioMasher pestle. Fragment size analysis of 0.01-0.5 ng of DNA was then performed using an Agilent FemtoPulse. High molecular weight (HMW) DNA was extracted using the Qiagen MagAttract HMW DNA extraction kit. Low molecular weight DNA was removed from a 200-ng aliquot of extracted DNA using 0.8X AMpure XP purification kit prior to 10X Chromium sequencing; a minimum of 50 ng DNA was submitted for 10X sequencing. HMW DNA was sheared into an average fragment size between 12–20 kb in a Megaruptor 3 system with speed setting 30. Sheared DNA was purified by solid-phase reversible immobilisation using AMPure PB beads with a 1.8X ratio of beads to sample to remove the shorter fragments and concentrate the DNA sample. The concentration of the sheared and purified DNA was assessed using a Nanodrop spectrophotometer and Qubit Fluorometer and Qubit dsDNA High Sensitivity Assay kit. Fragment size distribution was evaluated by running the sample on the FemtoPulse system.

### Sequencing

Pacific Biosciences HiFi circular consensus and 10X Genomics Chromium read cloud sequencing libraries were constructed according to the manufacturers’ instructions. Sequencing was performed by the Scientific Operations core at the Wellcome Sanger Institute on Pacific Biosciences SEQUEL II and Illumina NovaSeq 6000 instruments. Hi-C data were generated from head tissue of idGymRotn1 using the Arima Hi-C+ kit and sequenced on a NovaSeq 6000 instrument.

### Genome assembly

Assembly was carried out with Hifiasm ( Cheng *et al.*, 2021); haplotypic duplication was identified and removed with purge_dups ( Guan *et al.*, 2020). One round of polishing was performed by aligning 10X Genomics read data to the assembly with longranger align, calling variants with freebayes ( Garrison & Marth, 2012). The assembly was then scaffolded with Hi-C data ( Rao *et al.*, 2014) using SALSA2 ( Ghurye *et al.*, 2019). The assembly was checked for contamination as described previously ( Howe *et al.*, 2021). Manual curation ( Howe *et al.*, 2021) was performed using HiGlass ( Kerpedjiev *et al.*, 2018) and Pretext. The mitochondrial genome was assembled using MitoHiFi ( Uliano-Silva *et al.*, 2021), which performed annotation using MitoFinder ( Allio *et al.*, 2020). The genome was analysed and BUSCO scores generated within the BlobToolKit environment ( Challis *et al.*, 2020). Table 3 contains a list of all software tool versions used, where appropriate.

**Table 3.** Software tools used.

Software tool	Version	Source
Hifiasm	0.15.1	Cheng *et al.*, 2021
purge_dups	1.2.3	Guan *et al.*, 2020
SALSA2	2.2	Ghurye *et al.*, 2019
longranger align	2.2.2	https://support.10xgenomics.com/ genome-exome/software/pipelines/latest/ advanced/other-pipelines
freebayes	1.3.1-17-gaa2ace8	Garrison & Marth, 2012
MitoHiFi	2.0	Uliano-Silva *et al.*, 2021
HiGlass	1.11.6	Kerpedjiev *et al.*, 2018
PretextView	0.2.x	https://github.com/wtsi-hpag/PretextView
BlobToolKit	3.0.5	Challis *et al.*, 2020

### Ethics/compliance issues

The materials that have contributed to this genome note have been supplied by a Darwin Tree of Life Partner. The submission of materials by a Darwin Tree of Life Partner is subject to the Darwin Tree of Life Project Sampling Code of Practice. By agreeing with and signing up to the Sampling Code of Practice, the Darwin Tree of Life Partner agrees they will meet the legal and ethical requirements and standards set out within this document in respect of all samples acquired for, and supplied to, the Darwin Tree of Life Project. Each transfer of samples is further undertaken according to a Research Collaboration Agreement or Material Transfer Agreement entered into by the Darwin Tree of Life Partner, Genome Research Limited (operating as the Wellcome Sanger Institute), and in some circumstances other Darwin Tree of Life collaborators.

## Data availability

European Nucleotide Archive: Gymnosoma rotundatum. Accession number PRJEB46301; https://identifiers.org/ena.embl/PRJEB46301.

The genome sequence is released openly for reuse. The *G. rotundatum* genome sequencing initiative is part of the Darwin Tree of Life (DToL) project. All raw sequence data and the assembly have been deposited in INSDC databases. The genome will be annotated and presented through the Ensembl pipeline at the European Bioinformatics Institute. Raw data and assembly accession identifiers are reported in Table 1.

## Author information

Members of the Natural History Museum Genome Acquisition Lab are listed here: https://doi.org/10.5281/zenodo.5746819.

Members of the Darwin Tree of Life Barcoding collective are listed here: https://doi.org/10.5281/zenodo.5744972.

Members of the Wellcome Sanger Institute Tree of Life programme are listed here: https://doi.org/10.5281/zenodo.6125027.

Members of Wellcome Sanger Institute Scientific Operations: DNA Pipelines collective are listed here: https://doi.org/10.5281/zenodo.5746904.

Members of the Tree of Life Core Informatics collective are listed here: https://doi.org/10.5281/zenodo.6125046.

Members of the Darwin Tree of Life Consortium are listed here: https://doi.org/10.5281/zenodo.5638618.
