# Supplementary material for: The genome sequence of Gymnosoma rotundatum (Linnaeus, 1758), a parasitoid ladybird fly
Source: Wellcome Open Res. 2022 Mar 21;7:104. [Version 1] doi: 10.12688/wellcomeopenres.17782.1 (PMC8987346; doi:10.12688/wellcomeopenres.17782.1)
Supplement: Peer review history

# Peer review: Reviewer response for version 1

**DOI:** 10.21956/wellcomeopenres.19681.r49370

**Published:** 2022-04-06

**Authors:** Ken Kraaijeveld

**Affiliations:** 1 Faculty of Earth and Life Sciences, Institute of Ecological Science, Animal Ecology, Vrije University Amsterdam, Amsterdam, Netherlands Antilles

This paper presents the genome of a Tachinid fly, sequenced and assembled using state-of-the-art techniques. I have very little to add.

 In the introduction, some natural history information is given, but the reason for picking this species for genome sequencing is not discussed. The fact that the huge Tachnidae group is poorly represented among parasitoid insects with genome sequences available might be mentioned. Parasitoids are important model systems for various research topics as well as biological control agents. ^1^. However, genomics resources are limited, except for parasitoid wasps. ^2^

 As the authors are undoubtedly aware, it would have been nice to have some (short-read) data from a female of the same species. That would allow identification of Y-chromosomal contigs, as these would have very few of the female reads mapping to them.

Are sufficient details of methods and materials provided to allow replication by others?

Yes

Is the rationale for creating the dataset(s) clearly described?

No

Are the datasets clearly presented in a useable and accessible format?

Yes

Are the protocols appropriate and is the work technically sound?

Yes

Reviewer Expertise:

evolutionary genomics

I confirm that I have read this submission and believe that I have an appropriate level of expertise to confirm that it is of an acceptable scientific standard.

## References

[1] : Next-generation biological control: the need for integrating genetics and genomics. *Biol Rev Camb Philos Soc* .95(6) : 10.1111/brv.12641 1838-1854 10.1111/brv.12641 32794644PMC7689903

[2] : Genomic Resources for Goniozus legneri, Aleochara bilineata and Paykullia maculata, Representing Three Independent Origins of the Parasitoid Lifestyle in Insectsdust. *G3 Genes,Genomes,Genetics* .2019;9(4) : 10.1534/g3.119.300584 10.1534/g3.119.300584 PMC646941630705120

# Peer review: Reviewer response for version 1

**DOI:** 10.21956/wellcomeopenres.19681.r49368

**Published:** 2022-03-22

**Authors:** Jaakko L.O. Pohjoismäki

**Affiliations:** 1 Department of Environmental and Biological Sciences, University of Eastern Finland, Joensuu, Finland

Thank you for the opportunity to review the data note manuscript by Matthew Smith *et al.* on the genome sequence of *Gymnosoma rotundatum* (Diptera: Tachinidae). The approach taken by DToL can be seen as a benchmark for many other genome sequencing initiatives for providing a state-of-the-art, chromosomally scaffolded reference genome for the species. I am not sure how many exists for true flies, but to my knowledge this is the first for the family of Tachinidae (parasitic flies) and certainly the first for the subfamily of bug killing flies (Phasiinae). I have only few minor suggestions to improve the content of the manuscript.

 Title: “parasitoid ladybird fly” is a bit awkward. True that it refers to the vernacular (although not widely adopted) English name of the species, but it also might mislead a quick reader to think of ladybird beetles (Coccinellidae). I would suggest something in the line of “The genome sequence of a bug killing fly, *Gymnosoma rotudatum* (Linnaeus, 1758) (Diptera, Tachinidae)”. Spelling out the family name will help to find the paper for those who are interested in tachinids in general but are unfamiliar with the genus.

**Background:**

 Nit-picking, but I would put the size of *G. rotundatum* to 5-9 mm. As of note, there are taxonomic uncertainties in the genus due to the morphological variation and DNA barcode sharing (see DOI: 10.1111/syen.12450), for which the reference genome will probably bring more resolution. Fortunately, the specimen illustrated in the Figure 1. is photographed in a way that it shows enough detail to convince me that it really represents our current understanding of the taxon under the name *Gymnosoma rotundatum*. Taxonomically the publication is also on safe grounds as if there are synonyms in the future, *G. rotundatum* is the oldest name in the genus. There is also a comprehensive host catalogue for Palearctic Tachinidae here: http://www.nadsdiptera.org/Tach/WorldTachs/CatPalHosts/Home.html, which could be cited for the host records. As there are many different host species, they could be summed just as “suitable sized shield bugs (Pentatomidae) in the fly’s habitat”.

**Genome sequence report:**

 You might be able to infer the existence of a Y-chromosome indirectly (most calyptrate flies follow XY-system of sex determination) by looking at the existence of the dominant male-determining factor in your sequence data (e.g. *PLoS Biol.* **13**(4), e1002078). The fact that you have a haploid X, strongly suggests the XY-male type as there are other examples of Oestroidea with homomorphic sex chromosomes ( https://doi.org/10.1038/s41598-020-72880-0).

 Figs. 2-5. Looks very good. I especially enjoyed the chromosome-level mapping in Fig. 5. and its interactive version. Table 2: It is good to note that the fact that X chromosome is quite small compared to the others, is in line with some Calliphoridae (doi: 10.3897/CompCytogen.v9i1.8671).

 Sample acquisition & nucleic acids extraction: Collection date missing. Specimens in plural, are there others? No RNA was extracted? Was the whole specimen destroyed in the DNA extractions or is there some reference tissue left? Where (and how) is this stored and how can it be located? If the reference specimen is still existing, describe all associated labels. For later morphological analysis it would be great to preserve at least the male genitalia as a voucher. The voucher should be placed in a public collection. I assume that this is probably for all specimens in DToL but needs to be described here.

 Ethics/compliance: Was a collection permit required for the Hartslock Reserve? If so, please provide details.

Are sufficient details of methods and materials provided to allow replication by others?

Yes

Is the rationale for creating the dataset(s) clearly described?

Yes

Are the datasets clearly presented in a useable and accessible format?

Yes

Are the protocols appropriate and is the work technically sound?

Yes

Reviewer Expertise:

molecular biology, genetics, population genetics, evolution, Diptera taxonomy

I confirm that I have read this submission and believe that I have an appropriate level of expertise to confirm that it is of an acceptable scientific standard.

## References

[1] : The genomes of a monogenic fly: views of primitive sex chromosomes. *Scientific Reports* .2020;10(1) : 10.1038/s41598-020-72880-0 10.1038/s41598-020-72880-0 32978490PMC7519133

[2] : The thin red line between species – genomic differentiation ofGymnosoma Meigen, a taxonomically challenging genus of parasitoid flies (Diptera: Tachinidae). *Systematic Entomology* .2021;46(1) : 10.1111/syen.12450 96-110 10.1111/syen.12450

[3] : Numerous transitions of sex chromosomes in Diptera. *PLoS Biol* .2015;13(4) : 10.1371/journal.pbio.1002078 e1002078 10.1371/journal.pbio.1002078 25879221PMC4400102

[4] : Caroli Linnaei...Systema naturae per regna tria naturae :secundum classes, ordines, genera, species, cum characteribus, differentiis, synonymis, locis. 1758; 10.5962/bhl.title.542 10.5962/bhl.title.542

[5] : Comparative study of mitotic chromosomes in two blowflies, Luciliasericata and L.cluvia (Diptera, Calliphoridae), by C- and G-like banding patterns and rRNA loci, and implications for karyotype evolution. *Comp Cytogenet* .2015;9(1) : 10.3897/CompCytogen.v9i1.8671 103-18 10.3897/CompCytogen.v9i1.8671 25893078PMC4387384
